# Impact of Comorbid Alcohol Use Disorder on Health-Related Quality of Life Among Patients With Depressive Symptoms

**DOI:** 10.3389/fpsyt.2021.688136

**Published:** 2021-10-08

**Authors:** Kaisa E. Luoto, Lars H. Lindholm, Antti Koivukangas, Antero Lassila, Harri Sintonen, Esa Leinonen, Olli Kampman

**Affiliations:** ^1^Department of Psychiatry, Faculty of Medicine and Health Technology, Tampere University, Tampere, Finland; ^2^Department of Psychiatry, Hospital District of South Ostrobothnia, Seinäjoki, Finland; ^3^Department of Psychiatry, Tampere University Hospital, Pirkanmaa Hospital District, Tampere, Finland; ^4^Department of Public Health, University of Helsinki, Helsinki, Finland

**Keywords:** depression, alcohol use disorder, quality of life, behavioral activation, motivational interviewing, comorbidity

## Abstract

**Background and Aim:** In psychiatric clinical practice, comorbidity of depression and alcohol use disorder (AUD) is common. Both disorders have a negative impact on health-related quality of life (HRQoL) in general population. However, research on the impact of comorbid AUD on HRQoL among clinically depressed patients is limited. The purpose of this study was to explore the impact of a psychosocial treatment intervention on HRQoL for depressive patients in specialized psychiatric care with a special focus on the impact of AUD on HRQoL.

**Material and Methods:** Subjects were 242 patients of the Ostrobothnia Depression Study (ClinicalTrials.gov Identifier NCT02520271). Patients referred to specialized psychiatric care who scored at least 17 points on the Beck Depression Inventory at baseline and who had no psychotic disorders were included in the ODS. The treatment intervention in ODS comprised behavioral activation for all but began with motivational interviewing for those with AUD. HRQoL was assessed regularly during 24-month follow-up by the 15D instrument. In the present study, HRQoL of ODS patients with or without AUD was compared and the factors explaining 15D score analyzed with a linear mixed model. In order to specify the impact of clinical depression on HRQoL during the early phase of treatment intervention, a general population sample of the Finnish Health 2011 Survey was used as an additional reference group.

**Results:** HRQoL improved among all ODS study sample patients regardless of comorbid AUD during the first year of follow-up. During 12–24 months of follow-up the difference between groups was seen as HRQoL continued to improve only among the non-AUD patients. A combination of male gender, anxiety disorder, and AUD was associated with the poorest HRQoL in this sample. In combined sample analyses with the reference group, clinical depression had an impact on HRQoL in short-term follow-up regardless of the treatment intervention.

**Conclusions:** This study suggests that, in terms of improvement in HRQoL, the heterogenous group of depressive patients in specialized psychiatric care can be successfully treated with behavioral activation in combination with motivational interviewing for those with AUD.

**Clinical Trial Registration:** ClinicalTrials.gov Identifier: NCT02520271. Ostrobothnia Depression Study (ODS). A Naturalistic Follow-up Study on Depression and Related Substance Use Disorders. (2015). Available online at: https://clinicaltrials.gov/ct2/show/NCT02520271.

## Introduction

Patients with concurrent psychiatric and substance use disorder, i.e., dual diagnosis, are common in specialized psychiatric care, comorbid depression and alcohol use disorder (AUD) being one typical combination (1). Treatment of this dual pathology poses many challenges due, for example, to poorer treatment compliance and poorer effect of medications (2). Integrated treatment, i.e., psychiatric and substance use disorder managed in the same treatment facility, has been proposed for dual pathologies but more studies are needed on the effectiveness of these strategies in practice (3).

There is mounting evidence of the benefits of evidence-based treatment methods, such as behavioral activation (BA) (4) and motivational interviewing (MI) (5, 6), among dual diagnosed patients. A meta-analysis concerning the treatment of comorbid depression and AUD with combined cognitive behavioral therapy and MI showed a significant effect in treatment outcomes compared to treatment as usual (7). Promising results from an integrated care pathway for concurrent major depressive disorder and AUD have been reported in terms of reduction of alcohol consumption and alleviation of depressive symptoms (8).

The negative impact of psychiatric disorders on Health-Related Quality of Life (HRQoL) is well-established in general population and depressive and anxiety disorders seem to have the greatest impact (9–11). Heavy drinking and alcohol dependence have also been shown to affect HRQoL although the impact is smaller than that of mood and anxiety disorders (10–13).

The research on the impact of comorbid AUD on HRQoL among depressed patients is limited, likewise on the impact of psychiatric treatment interventions on these patients' HRQoL. To the best of our knowledge, only one study has so far explored quality of life as a treatment outcome during psychiatric treatment in patients with major depressive disorder and comorbid AUD (14). Danovitch et al. made a *post-hoc* analysis of the Sequenced Treatment Alternatives to Relieve Depression (STAR*D) study, where the treatment intervention was SSRI medication of 12–14 weeks for psychiatric outpatients (15). They analyzed a sample of 2,280 outpatients with major depressive disorder of whom 121 had comorbid AUD and used quality of life as one outcome measure. The authors conclude that, contrary to their expectations, no significant difference in quality of life was observed between AUD and non-AUD patients during follow-up.

The Ostrobothnia Depression Study (ODS) in Finland (16) was targeted at clinically depressed patients treated in specialized psychiatric care. The ODS intervention included a systematic evaluation of patients' total symptoms and possible comorbid substance abuse at the beginning of treatment and the components of the treatment intervention were BA for depression and MI for substance abuse. A more detailed description of the ODS intervention and its beneficial effects on depressive symptoms and functional recovery has been reported elsewhere (17).

In the ODS sample, virtually all patients with comorbid substance abuse were using alcohol. This gave us a chance to address the impact of comorbid AUD on HRQoL in this naturalistic sample. The present study aimed to explore the factors explaining HRQoL during the 2 years of follow-up in patients treated with the ODS intervention. Specifically, we aimed to learn more about the impact of comorbid AUD on HRQoL of clinically depressed patients and to specify the impact of clinical depression on HRQoL during the early phase of treatment.

## Materials and Methods

### ODS Study Sample and Procedures

The ODS was a benchmark controlled trial that aimed to assess the impact of clinical intervention in routine settings. The detailed protocol of the ODS study is previously reported elsewhere (17) and the study is registered in ClinicalTrials.gov with identifier NCT02520271 (16).

Patients (*n* = 242, age range 18–64, 61.2% female) were recruited from the natural patient flow of the participating units in the Department of Psychiatry in the South Ostrobothnia Hospital District, Finland during the period 2009–2013. The inclusion criteria were age ≥18 years and Beck Depression Inventory (BDI) score corresponding to at least moderate-level depression (BDI score ≥17) at baseline (18). Finnish translation of BDI has been validated by defining cut-off points and predictive values (19). Patients with psychotic or organic brain disorders (ICD-10, F20-29, or F00-09) were excluded.

Psychiatric diagnoses of the ODS study patients were then assessed according to the Mini-International Neuropsychiatric Interview (M.I.N.I.) (20) at baseline. Due to some incomplete baseline information, M.I.N.I. was available for 219 patients. The most common diagnosis was major depressive disorder (*n* = 194, 80.2%). All diagnoses made at baseline are presented in Table 1, which shows the variation in comorbid psychiatric problems in addition to the depressive symptoms. The mean age of the ODS study patients was 38.8 years (SD 12.2). The mean clinical symptom scores at baseline were as follows: BDI 27.9 (SD 7.3), Montgomery-Åsberg Depression Rating Scale (MADRS) (21) 23.2 (SD 6.7) and Alcohol Use Disorders Identification Test (AUDIT) (22) 10.7 (SD 9.9). Most patients (*n* = 203, 84.9%) were taking anti-depressive medication (fluoxetine equivalent daily dose; median 25.0, IQR 25.0) and 66 (27.3%) were taking adjuvant antipsychotic medication (chlorpromazine equivalent daily dose; median 62.5, IQR 93.8).

**Table 1:** All psychiatric diagnoses of the Ostrobothnian Depression Study patients (*n* = 219) at baseline according to the Mini-International Neuropsychiatric Interview (M.I.N.I.).

**ICD-10**	**Diagnosis according to M.I.N.I**.	***N* (%)**
F32, F33	Major depressive disorder	194 (88.9)
F34	Dysthymia	17 (7.8)
F31	Hypomania or mania (lifetime)	27 (12.3)
F41.2	Generalized anxiety disorder	69 (31.5)
F41.0	Panic disorder	50 (22.8)
F40.0	Agoraphobia	37 (16.9)
F40.1	Social anxiety disorder	43 (19.6)
F42	Obsessive-compulsive disorder	17 (7.8)
F43	Post-traumatic stress disorder	18 (8.2)
F10	Alcohol-related disorder	70 (40)
F1x	Substance-related disorder	11 (5.2)
F50.2	Bulimia	7 (3.2)

Among other measures during the ODS, the HRQoL of the study patients was rated at baseline and at three follow-up points (6, 12, and 24 months) by 15D, which is a comprehensive 15-dimensional instrument for measuring HRQoL among adults (23). 15D is a self-rated questionnaire that forms a 15D score on a scale 0–1 (1 meaning the best possible result) to express the overall HRQoL of the individual. A difference of ≥0.015 is considered a clinically significant difference in 15D (24). 15D score at baseline was available for 221 patients. 15D score during follow-up was available for patients continuing in the study at the follow-up points (6, 12, and 24 months, *n* = 145, 132, and 101, respectively).

When entering the study, the ODS patients were screened with AUDIT and categorized into subgroups according to their baseline AUDIT score using a cut-off of 10 points. Finnish translation of AUDIT has been validated by defining cut-off points and predictive values (25). Patients with baseline AUDIT > 10 (*n* = 99, 40.9%; 61 males) were categorized into subgroup as having comorbid AUD and patients with baseline AUDIT <10 (*n* = 131, 54.1%; 33 males) were categorized into subgroup as non-AUD patients. The groups were defined using AUDIT limit >10 instead of the diagnostic criteria of alcohol abuse according to M.I.N.I. This was due to the assumption that current AUDIT score better reflects the level of current alcohol consumption and is therefore more likely to indicate changes in HRQoL. According to M.I.N.I., lifetime alcohol use disorder was diagnosed in 65 (65.7%) of the AUD group patients.

### Comparisons in the Present Study

In the present study, the AUD and non-AUD groups of the ODS study sample were compared. Independent samples *t-*test was used to compare 15D scores between the AUD and non-AUD groups. A linear mixed model for repeated measures was used to analyze factors explaining 15D score at 24-month follow-up. The model was adjusted for age, gender, baseline MADRS score, and any anxiety disorder comorbidity diagnosis. AUD group (non-AUD or AUD patient), time (from baseline to 24 months), and an interaction term time*AUD group (combined effect of time and AUD group) were used as explanatory variables.

In addition, the Finnish Health 2011 Survey (H2011) sample (BRIF8901; www.terveys2011.info) (26) was used as an additional reference group in order to specify the impact of clinical depression on HRQoL during the early phase of treatment. The H2011 is a general population sample comprising 4,323 individuals (56% female) aged at least 29 years (mean age 56, SD 14).

Two separate analyses were conducted on the combined sample of ODS and H2011 to explore the impact of clinical depression on HRQoL during the first 6 months of treatment. The first model comprised the H2011 sample combined with the ODS sample data from baseline. The second model comprised the H2011 sample combined with the ODS sample data from the 6-month follow-up point. A Tobit regression model was used due to the relatively large number of patients with the best possible result (15D = 1 in 13.8% of all observed) at baseline in these combined samples. Due to the non-linear association between the 15D score and AUDIT/BDI scores in the explorative analysis, the square transformations of both BDI and AUDIT were added to the models including the reference sample data. However, this was not necessary for the analyses of the ODS sample alone. The models were adjusted for age, gender, BDI score, AUDIT score, and comorbid anxiety disorder at baseline. Belonging to the ODS or H2011 patient group was used as the explanatory factor in both analyses.

### Other Considerations

The ODS study protocol was approved by the Hospital District of South Ostrobothnia ethics committee (reference number EVO1114). Written informed consent was obtained from all participants. Patients received written information and they had the opportunity to ask for additional information during the study and also to withdraw from the study at any time without being excluded from treatment. The use of the H2011 data in this study was approved by the National Institute of Health and Welfare of Finland.

In power analysis between the ODS and Finnish Health Survey samples, the independent analyses were able to detect a 0.017 point mean difference in 15D total score with a power of 0.8 and a type I error probability of 0.05. The level of statistical significance in all analyses was set at *p* < 0.05. Calculations were performed with PS Power and Sample Size Calculator (27), SAS/STAT [comparative analyses between ODS and H2011 samples (28)] and SPSS for Apple Macintosh versions 24 and 25 (29).

## Results

### ODS Study Sample Analyses

**Figure 1:**
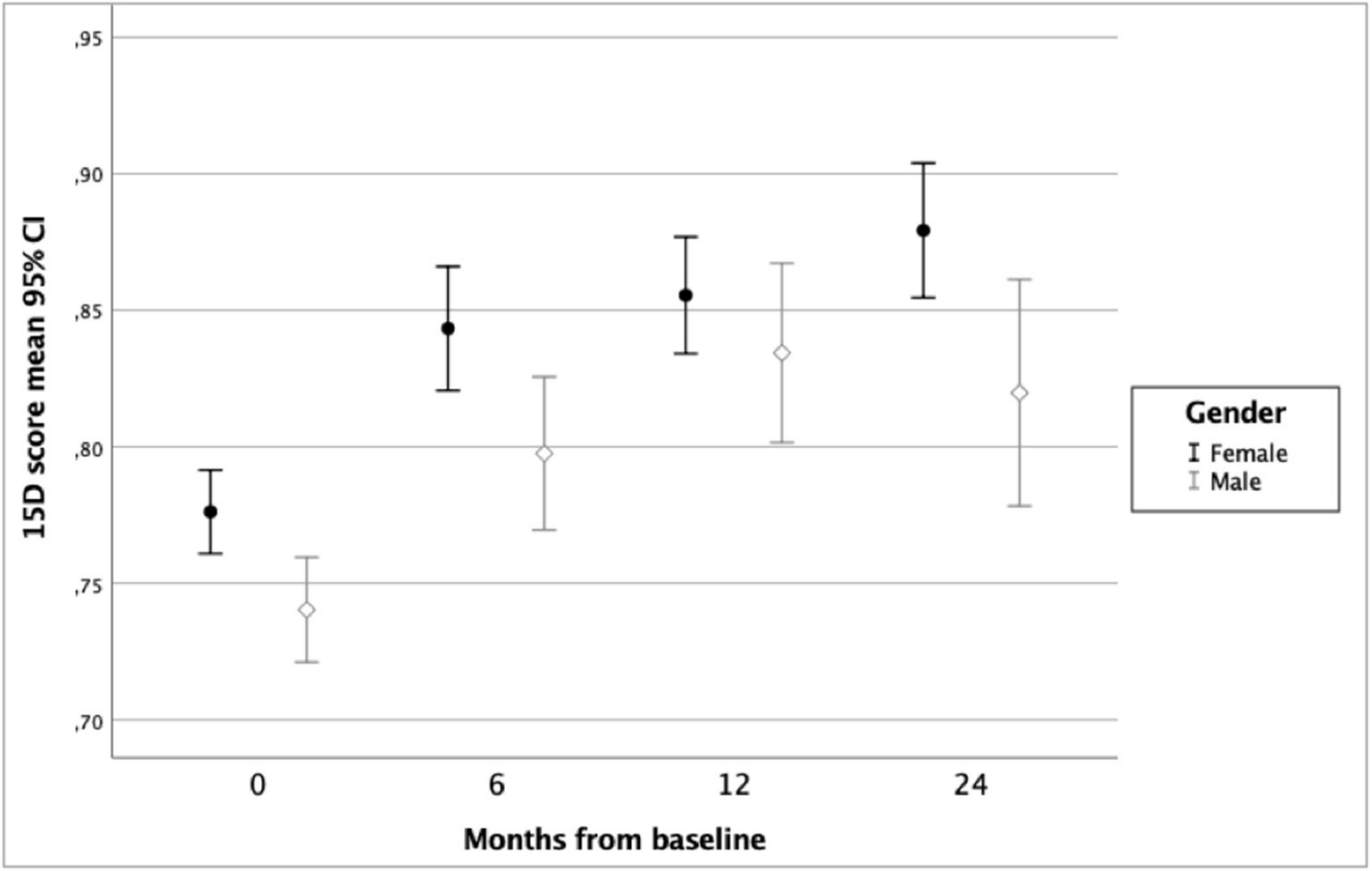
Health-related quality of life of ODS study patients according to 15D score and gender.

**Figure 2:**
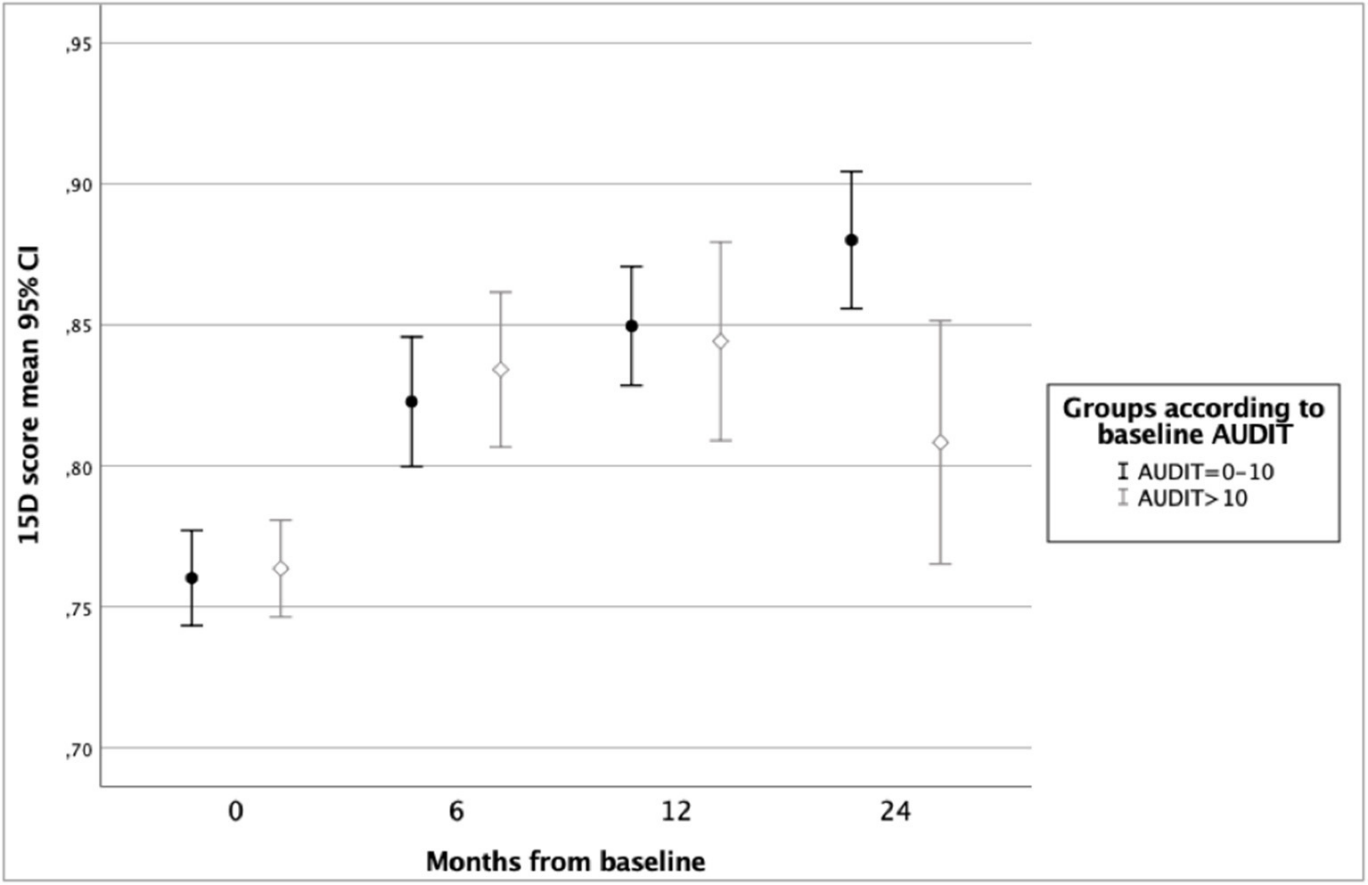
Health-related quality of life of ODS study patients according to 15D score and baseline Alcohol Use Disorders Identification Test (AUDIT) score.

**Figure 3:**
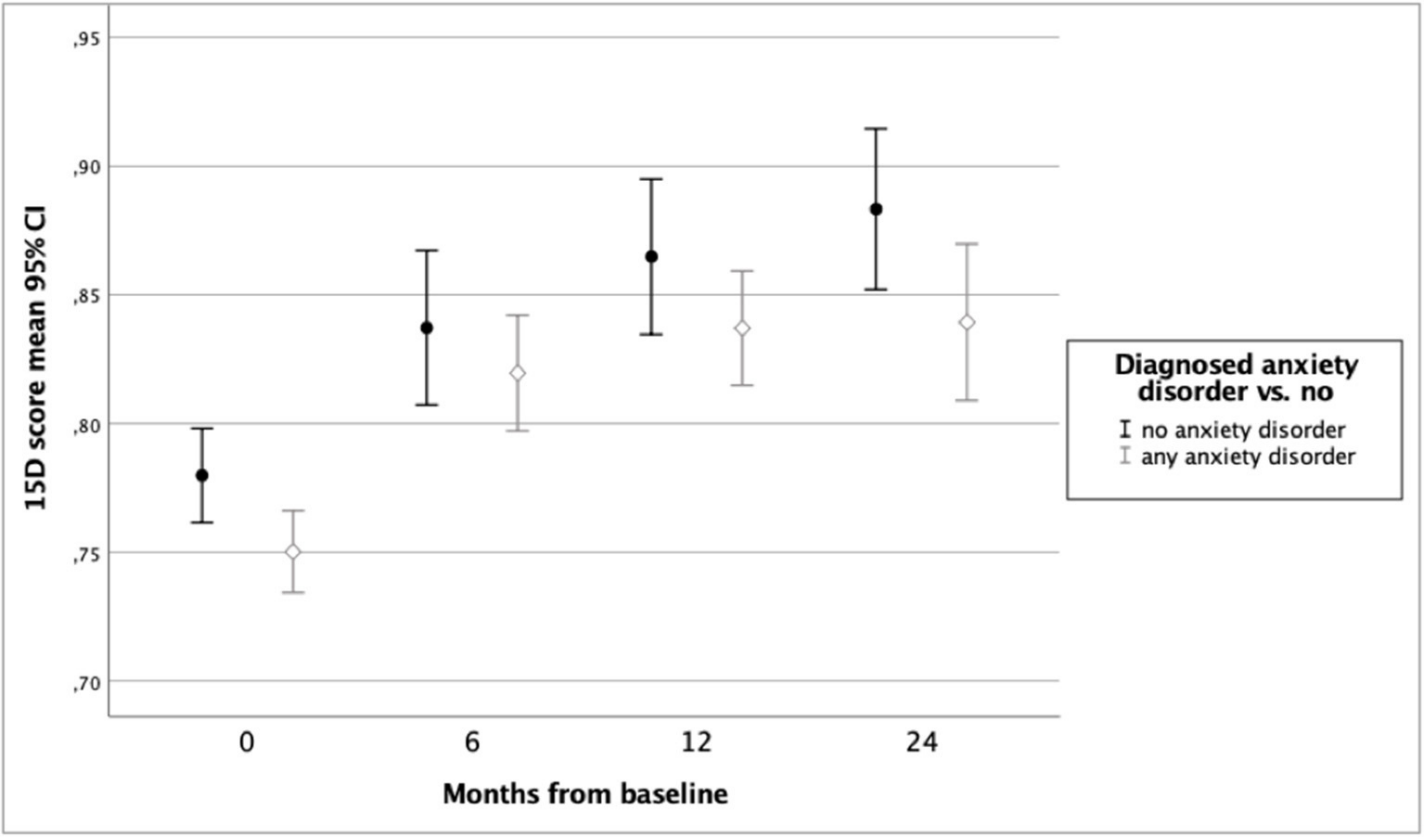
Health-related quality of life of ODS study patients according to 15D score and baseline anxiety disorder.

In the ODS study sample (*n* = 242), mean improvement in 15D score from baseline to 6 months was 0.066 (*p* < 0.001), from 6 to 12 months 0.022 (*p* = 0.001) and from 12 to 24 months 0.007 (*p* = 0.19). Variation and changes in HRQoL during 24 months of follow-up according to groups by gender, baseline alcohol use disorder, and diagnosis of any anxiety disorder are shown in Figures 1–3.

Table 2 presents HRQoL during 24 months of follow-up among both AUD and non-AUD groups. HRQoL improved in both groups until 1 year of follow-up and in the non-AUD group until two years of follow-up. In AUD group, HRQoL declined from 12 to 24 months of follow-up. Therefore, a statistically significant difference in HRQoL between AUD and non-AUD patients was found at 24-month follow-up (mean 15D score 0.880 vs. 0.808, *p* = 0.002). Among the AUD patients (*n* = 99), AUDIT scores decreased significantly during the first 6 months of follow-up [20.8 (SD7.2) at baseline vs. 11.8 (SD 6.0) at 6 months, *p* ≤ 0.001].

**Table 2:** Health-related quality of life according to 15D score during 24 months of follow-up among Ostrobothnian Depression Study patients with or without baseline alcohol use disorder.

**Follow-up point**	**Group^a^**	***n***	**15D score^b^ (SD)**	***p* ^c^**
Baseline	Non-AUD	131	0.760 (0.098)	0.790
	AUD	90	0.765 (0.082)	
6 months	Non-AUD	100	0.823 (0.116)	0.561
	AUD	45	0.834 (0.092)	
12 months	Non-AUD	93	0.850 (0.102)	0.788
	AUD	39	0.844 (0.109)	
24 months	Non-AUD	70	0.880 (0.102)	0.002
	AUD	31	0.808 (0.118)	

a *Patient group according to baseline Alcohol Use Disorders Identification Test (AUDIT) score (non-AUD when AUDIT ≤ 10)*.

b *Health-related Quality of Life measured by 15D instrument, mean score on a scale 0–1*.

c *T-test between AUDIT-based patient groups*.

Table 3 presents the results of a linear mixed model for repeated measures of HRQoL during 24 months of follow-up and including possible confounding factors. In this model, female gender, belonging to the non-AUD group, not having a comorbid anxiety disorder, and having a lower baseline score on MADRS were found to be significant explanatory variables for better HRQoL. The estimates of 15D showed that HRQoL improved over 24 months in the total sample. Belonging to the AUD group was only a trend level explanatory factor, whereas the variables time (for follow-up) and the interaction term time*AUD were significant predictors, indicating better HRQoL in non-AUD group toward the end of follow-up.

**Table 3:** The results of linear mixed model for repeated measures of health-related quality of life of Ostrobothnian Depression Study patients measured by 15D instrument and including possible confounding factors.

		**95% CI**			
**Explanatory variable**	**Estimate^a^**	**Lower**	**Upper**	***t***	***p***
Female gender	0.025	0.002	0.048	2.115	0.036
Age	−0.001	−0.002	5.313E-05	−1.846	0.066
Baseline MADRS^b^ score	−0.005	−0.007	−0.003	−6.051	<0.001
Baseline AUDIT^c^ ≤ 10 points	0.038	−0.002	0.078	1.866	0.064
No anxiety disorder	0.027	0.006	0.048	2.506	0.013
**Time (among all patients, 24 months as reference)**					
Baseline	−0.119	−0.140	−0.098	−11.280	<0.001
6 months	−0.054	−0.074	−0.034	−5.282	<0.001
12 months	−0.025	−0.043	−0.008	−2.909	0.004
24 months	0				
Time*AUD group^d^ (combined effect of time and AUD, 24 months as reference)					
Baseline	−0.062	−0.100	−0.025	−3.308	0.001
6 months	−0.065	−0.102	−0.029	−3.536	0.001
12 months	−0.048	−0.079	−0.017	−3.055	0.003
24 months	0				

a *Interpretation of factor estimates: a positive estimate indicates higher 15D score in women, in patients with baseline AUDIT <10, and in patients with no anxiety disorder. A negative estimate in factors time and time*

* *AUD indicates lower 15D score compared to the 24-month follow-up point*.

b *Montgomery-Åsberg depression rating scale*.

c *Alcohol use disorders identification test*.

d *Non-AUD group when AUDIT ≤10 and AUD group when AUDIT > 10*.

### Combined Sample of ODS and H2011

In the combined sample, the factors explaining HRQoL in the first model (applying the ODS baseline data and H2011 data) were age, BDI, AUDIT scores, and comorbid anxiety disorder, all *p*-values < 0.0001. In the corresponding analysis with the 6-month follow-up data of the ODS sample together with H2011 data, all above variables and also belonging to the ODS study group (i.e., being treated for depression in specialized psychiatric care) were found to be significant explanatory factors for poorer HRQoL (Table 4). With these combined sample analyses we demonstrated that clinical depression has an impact on HRQoL in short-term follow-up regardless of the treatment intervention.

**Table 4:** Results of the tobit regression model with the 6-month follow-up data of the Ostrobothnian Depression Study (ODS) sample together with Finnish Health 2011 Survey data.

**Explanatory variable**	**Coefficient**	**SE**	***p***
Constant	0.91	0.0063	<0.0001
ODS	0.053	0.0064	<0.0001
Age	−0.0014	0.000072	<0.0001
Gender	−0.0024	0.0020	0.223
BDI-21^a^ score	−0.10	0.00031	<0.0001
AUDIT-C^b^ score	0.0050	0.0011	<0.0001
Anxiety disorder	−0.030	0.0045	<0.0001
BDI_sq^c^	0.00011	0.000010	<0.0001
AUDIT_sq	−0.00046	0.00012	0.0001

a *Beck depression inventory*.

b *Alcohol use disorders identification test-concise*.

c *The square transformations of BDI and AUDIT*.

## Discussion

In this study of a naturalistic sample of clinically depressed patients in specialized psychiatric care, we explored the impact of an evidence-based treatment intervention in the HRQoL of depressive patients with or without comorbid AUD. We found that HRQoL improved among all patients (regardless of comorbid AUD) during the first year of follow-up. During the second year of follow-up, HRQoL improved among the non-AUD patients but decreased slightly among those with AUD at baseline. Thus, a group difference in HRQoL was seen at 2-year follow-up, when the patients with baseline AUD had poorer HRQoL than those without AUD at baseline. However, HRQoL of AUD group patients was still better at 24 months than it was at baseline. Moreover, a comorbid anxiety disorder was found to have a significantly negative impact on improvement in HRQoL. According to this study, the combination of male gender, comorbid anxiety disorder, and AUD was associated with the poorest HRQoL.

To specify the impact of clinical depression among other factors on overall HRQoL combined sample analysis including the general population sample H2011 and the ODS patient sample was performed. The predictors of poorer HRQoL in the combined sample were more severe depressive symptoms, anxiety, and comorbid alcohol use, which is in line with existing research concerning HRQoL in general population samples (9–11, 13). When ODS data at 6-month follow-up was used in the combined sample analysis, being treated for depression in specialized psychiatric care was found to be an independent explanatory factor for poorer HRQoL. This means that despite the alleviation of depressive symptoms during ODS treatment, the patients' HRQoL did not improve accordingly.

Many patients with major depressive disorder continue to experience impaired quality of life although the depressive symptoms diminish (30). A study exploring HRQoL among primary care depressive patients suggested that the patients had to be virtually free of symptoms of depression and anxiety before improvement in HRQoL could commence (31). As noted in the introduction, there is a scarcity of research on HRQoL as a treatment outcome among clinically depressed patients with AUD. According to the study by Danovitch et al. (14) severity of depressive symptoms was found to improve most during the intervention, whereas there was only a modest improvement in quality of life scores.

In our sample, at 24-month follow-up HRQoL of non-AUD patients was better than among those with AUD. It is possible that the impact of AUD becomes more prominent after the depressive symptoms diminish during treatment. Compared to depression and anxiety, it may take a longer time before substance abuse affects quality of life due, for example, to a long latency with heavy alcohol consumption in causing physical or social problems (11). As there was already a marked recovery from alcohol consumption in the AUD group during the first 6 months in ODS, factors other than the current alcohol consumption likely explain the group differences at 24 months in our study. For example, it is possible that the improvements following the intervention were less long-lasting among the patients with AUD. Since the ODS treatment intervention began with MI among the AUD group patients, it is not possible to differentiate which method (BA, MI, or a combination of these) was more beneficial in improving HRQoL among this subgroup.

Our sample of ODS patients is highly representative of an unselected patient population in psychiatric secondary services. Thus, the generalizability of the results among the real-life depressive patients can be considered good and the naturalistic setting and the heterogenous patient group can be considered as a strength when evaluating the treatment outcomes in routine clinical settings. However, the heterogeneity of the participants can also be considered as a limitation which may decrease the value of the study findings and may have an impact on the interpretation of the results.

The inclusion criteria for the study were based on BDI screening. However, the final diagnosis was set according to the structured diagnostic interview and about 90% of included patients were diagnosed with major depressive disorder. Harmful drinking was determined according to the AUDIT score which has been shown to have a good sensitivity in detecting current drinking problems. Different comorbid anxiety disorders are very common and often have a significant impact on the treatment response in depressive patients. Anxiety disorders were the most common comorbidities also among the ODS patient sample. About 12% of patients in our study had a previous episode of hypomania or mania according to MINI. However, the patients had depressive symptoms at the beginning of the ODS intervention and were therefore also included in the ODS study. The depressive episodes of unipolar and bipolar disorders were considered clinically comparable in this study.

As the study sample was a naturalistic patient sample within psychiatric secondary services, the patients took various combinations of psychiatric medications. There was skewness in the gender distribution between the AUD and non-AUD groups as there were more males in the AUD group. The relatively small size of the clinical sample due to drop-out during follow-up may also inhibit the generalizability of the results.

Our study adds to the positive evidence on treating patients with depression and comorbid alcohol use with simultaneous motivational interviewing and cognitive behavioral methods such as BA. Studies on treatment interventions for depressive patients often exclude patients with substance use disorders. However, these patients are quite common in clinical practice. Thus, research is needed on effective treatment methods for this subgroup of patients. Despite the rapid clinical symptom relief, full recovery from a depressive episode usually takes at least a year and changes in HRQoL also manifest relatively slowly. Therefore, studies with a longer follow-up are needed on this issue. More studies concentrating on patient centered outcomes, such as HRQoL or functional recovery, are needed to better inform future treatment strategies.

## Conclusions

Comorbid alcohol use problems and anxiety disorders should be taken into account in the early phase of treatment for depression since both are detrimental to quality of life among patients with clinical depression. Our study suggests that, in terms of improvement in HRQoL, the heterogenous group of depressive patients in specialized psychiatric care could be treated successfully with behavioral activation combined with motivational interviewing for those with AUD.

## Data Availability Statement

The raw data supporting the conclusions of this article will be made available by the authors, without undue reservation.

## Ethics Statement

The studies involving human participants were reviewed and approved by The Hospital District of South Ostrobothnia Ethics Committee (reference number EVO1114). The patients/participants provided their written informed consent to participate in this study.

## Author Contributions

KL: original draft, review and editing, and formal analysis. LL: review and editing. AK: conceptualization and review and editing. AL: conceptualization, project administration, and review and editing. HS: formal analysis and review and editing. EL: conceptualization and review and editing. OK: conceptualization, methodology, data curation, formal analysis, review and editing, and supervision. All authors contributed to the article and approved the submitted version.

## Funding

This work was supported by a grant from the Finnish Medical Foundation (grant number 4282) and the research fund of Hospital District of South Ostrobothnia.

## Conflict of Interest

HS is the developer of the 15D and obtains royalties from its electronic versions. The remaining authors declare that the research was conducted in the absence of any commercial or financial relationships that could be construed as a potential conflict of interest. The reviewer OL declared a shared affiliation, with no collaboration, with the author HS at the time of the review.

## Publisher's Note

All claims expressed in this article are solely those of the authors and do not necessarily represent those of their affiliated organizations, or those of the publisher, the editors and the reviewers. Any product that may be evaluated in this article, or claim that may be made by its manufacturer, is not guaranteed or endorsed by the publisher.
